# The Aza-Analogous Benzo[*c*]phenanthridine P8-D6 Acts as a Dual Topoisomerase I and II Poison, thus Exhibiting Potent Genotoxic Properties

**DOI:** 10.3390/molecules25071524

**Published:** 2020-03-27

**Authors:** Georg Aichinger, Falk-Bach Lichtenberger, Tamara N. Steinhauer, Inken Flörkemeier, Giorgia Del Favero, Bernd Clement, Doris Marko

**Affiliations:** 1 University of Vienna, Faculty of Chemistry, Department of Food Chemistry and Toxicology, Waehringerstr. 38, A-1090 Vienna, Austria; georg.aichinger@univie.ac.at (G.A.); falk.lichtenberger@charite.de (F.-B.L.); giorgia.del.favero@univie.ac.at (G.D.F.); 2 Christian-Albrechts-University Kiel, Pharmaceutical Institute, Department of Pharmaceutical and Medicinal Chemistry, Gutenbergstraße 76, D-24118 Kiel, Germany; tsteinhauer@pharmazie.uni-kiel.de (T.N.S.); ifloerkemeier@pharmazie.uni-kiel.de (I.F.); bclement@pharmazie.uni-kiel.de (B.C.)

**Keywords:** chemotherapy, drug development, topoisomerase poisoning, dual topoisomerase inhibitor, genotoxicity, nuclear localization, confocal microscopy

## Abstract

The benzo[*c*]phenanthridine P8-D6 was recently found to suppress the catalytic activity of both human topoisomerase (Topo) I and II. Concomitantly, potent cytotoxic activity was observed in different human tumor cell lines, raising questions about the underlying mechanisms in vitro. In the present study, we addressed the question of whether P8-D6 acts as a so-called Topo poison, stabilizing the covalent Topo–DNA intermediate, thus inducing fatal DNA strand breaks in proliferating cells. In HT-29 colon carcinoma cells, fluorescence imaging revealed P8-D6 to be taken up by the cells and to accumulate in the perinuclear region. Confocal microscopy demonstrated that the compound is partially located inside the nuclei, thus reaching the potential target. In the “in vivo complex of enzyme” (ICE) bioassay, treatment of HT-29 cells with P8-D6 for 1 h significantly enhanced the proportion of Topo I and II covalently linked to the DNA in concentrations ≥1 µM, indicating effective dual Topo poisoning. Potentially resulting DNA damage was analyzed by single-cell gel electrophoresis (“comet assay”). Already at 1 h of incubation, significant genotoxic effects were observed in the comet assay in concentrations as low as 1 nM. Taken together, the present study demonstrates the high Topo-poisoning and genotoxic potential of P8-D6 in human tumor cells.

## 1. Introduction

**Figure 1:**
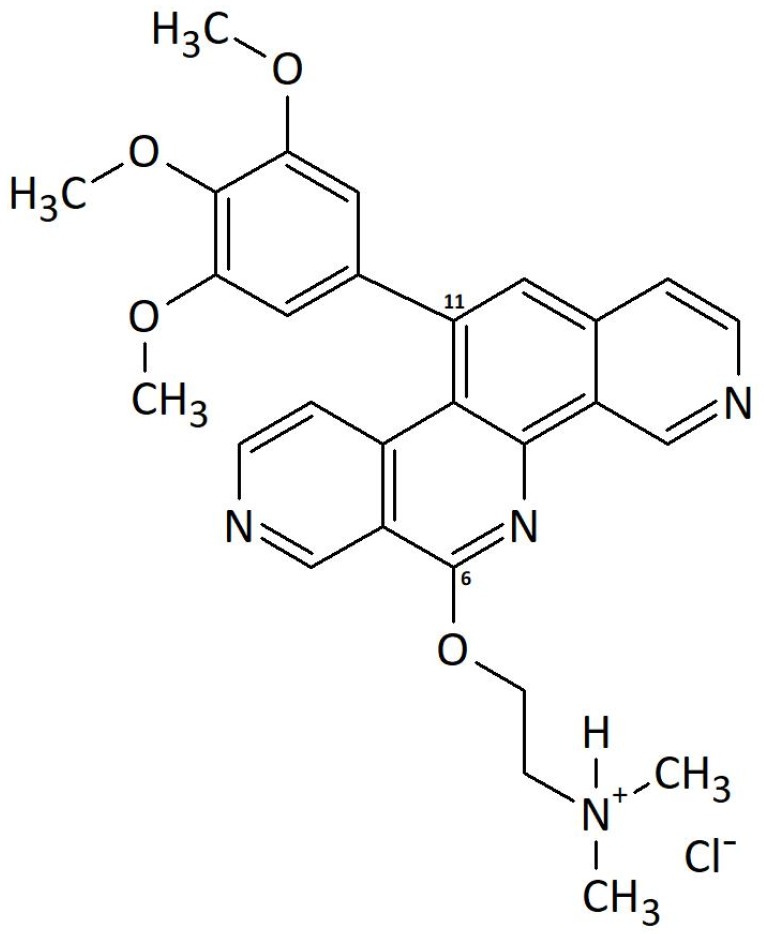
Chemical structure of P8-D6 (6-(*N,N*-dimethyl-2-aminoethoxy)-11-(3,4,5-trimethoxyphenyl)pyrido[3,4-*c*][1,9]phenanthroline hydrochloride).

Benzo[*c*]phenanthridines have a wide-ranging pharmacological spectrum of action. Especially cytotoxic, but also antimicrobial and anti-inflammatory effects have been previously described [1,2]. Naturally occurring substances of this drug class, such as fagaronine, have been described to inhibit topoisomerase I and II by stabilizing the covalent DNA–enzyme intermediate [3]. Due to poor yields and toxicological problems, synthesis of azo-analogous benzo[*c*]phenanthridines was previously optimized into a four step process [4,5]. The synthetic products were improved to enhance physicochemical properties and cytotoxicity. In the context of possible pharmaceutical applications, the compound P8-D6 (Figure 1) exerted the best properties in terms of high photostability, solubility, and cytotoxicity in our studies.

In order to predict effects and side effects of drugs, detailed knowledge about their mechanisms of action is of great importance. We recently described P8-D6 to exert strong cytotoxic, pro-apoptotic, and anti-leukemic effects due to its ability to inhibit human topoisomerase (Topo) isoforms I and II, thus acting as a so-called “dual Topo inhibitor” [5].

Topos represent well-established targets in chemotherapy and play a central role in the maintenance of DNA topology and prevention of torsion stress. Especially in metabolically active and proliferating cells, characteristics known for many tumor cells, high Topo expression is needed to allow, among other processes, the coiling and uncoiling processes that make DNA accessible to transcriptional and replicational enzymes [6,7]. In humans, two main types of Topos are expressed: Topo I relaxes supercoiled DNA by inducing a single-strand break. In contrast, the ATP-dependent Topo II isoforms covalently bind to the DNA as dimers, each monomer attached to the phosphate backbone of one strand in an intermediate form known as a “cleavable complex”. As a result, a transient DNA double-strand break is generated, enabling a second strand to pass through the gap, followed by religation of the DNA and the release of the Topo from covalent binding to the phosphate backbone [6,7,8]. This catalytic cycle allows interference at different steps. “Catalytic Topo inhibitors” prevent the formation of the complex between Topo and DNA or—in the case of Topo II—the binding of ATP, which prevents the induction of a strand break [9]. Of higher relevance regarding clinical applications are so-called “Topo poisons”, which exert a higher toxicity against tumor cells. Such compounds stabilize the cleavable complex and thus inhibit the release of the enzyme and the religation of the DNA, which leads to torsion stress and potential collisions of approaching replication forks with the trapped DNA–Topo intermediates, resulting in DNA damage and consequently in cell cycle arrest, the induction of apoptosis, and cell death [10]. Several Topo poisons have been approved for the chemotherapeutic treatment of different cancer types in recent decades, including the Topo I inhibitors camptothecin and topotecan [11] and the Topo-II-targeting drugs etoposide and amsacrin [10].

A novel, promising class of chemotherapeutics are dual Topo inhibitors, which inhibit both type I and II enzymes. The possibly lower resistance susceptibility when inhibiting both type I and II enzymes might represent the main benefit of the latter [12]. However, no dual Topo inhibitors have so far been approved for clinical application.

We recently demonstrated the high capability of P8-D6 to induce apoptosis and cell death in various cancer cell lines [5]. In the NCI-60 DTP Human Tumor Cell Line screening, the average growth inhibition to 50% (GI_50_) over all 60 human tumor cell lines was 49 nM, and P8-D6 was thus found to be more active than the natural alkaloids fagaronin and nitidine. This remarkable effect was also perceived in colon cancer cells, with an average GI_50_ value of 63 nM. We also reported its ability to inhibit human topoisomerase I and II under cell-free conditions, which is believed to be the main mechanism regarding its cytostatic effects. However, this hypothesis has yet to be confirmed in vitro and the exact mechanism of action has yet to be investigated.

Therefore, the aim of the present study was to confirm the suspected ability of P8-D6 to enter the nucleus, to interfere with topoisomerases, and thus to cause the induction of DNA strand breaks by stabilizing the cleavable Topo–DNA complex. For our experiments, we chose to use HT-29 colon carcinoma cells, a cell model that was used in several recent studies dealing with topoisomerases [13,14,15], to ensure the comparability with previous results.

## 2. Results

### 2.1. Localization of P8-D6 in Cellular Compartments

**Figure 2:**
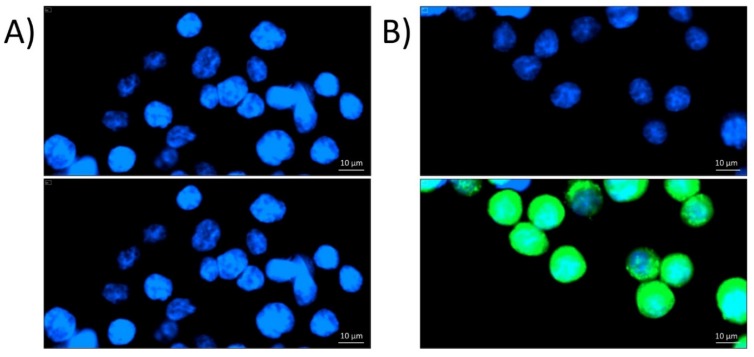
Fluorescence images of HT-29 cells after 1 h incubation with either the solvent control (**A**) or 50 µM P8-D6 (**B**), taken with a BioTekTM CytationTM3. The upper pictures show just the nuclear staining with Hoechst 33258, pictures at the bottom show a combined image of nuclear staining and P8-D6 auto-fluorescence at 525 nm.

**Figure 3:**
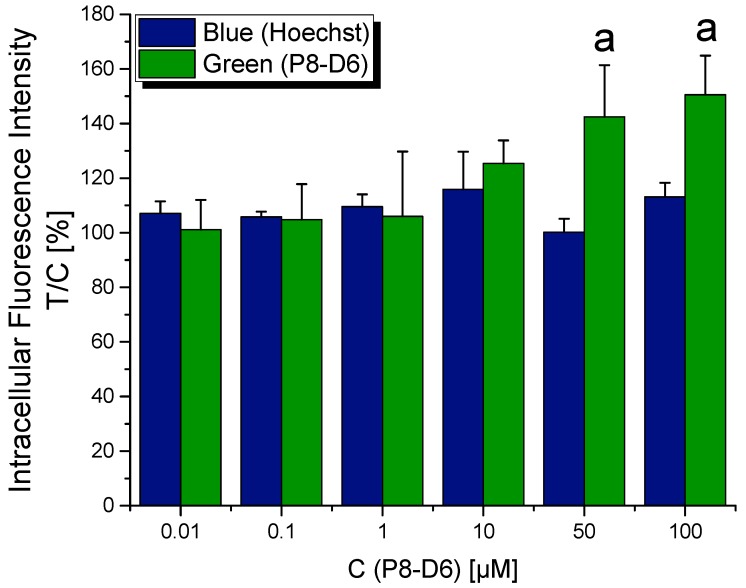
Quantified fluorescence signals of the stained DNA in HT-29 cells after an incubation of 1 h with P8-D6 at concentrations between 0.01 μM and 100 μM, expressed relative to the solvent control. Values are expressed as mean + SD of at least three independent biological experiments. Statistical significances (*p* < 0.05) were calculated by applying one-way ANOVA to green/blue test/control values, followed by Fisher’s Least Significant Difference (LSD) test, with “a” indicating a difference to the respective no-effect level with *p* < 0.05.

After 1 h of incubation, P8-D6 was found to be taken up by the cells and to accumulate in or around the nuclei when applied at concentrations of ≥10 µM (Figure 2). This was also reflected in the quantification results of intracellular green fluorescence, which was significantly enhanced in a concentration-dependent manner for cells incubated with P8-D6 at concentrations of ≥50 µM (Figure 3).

**Figure 4:**
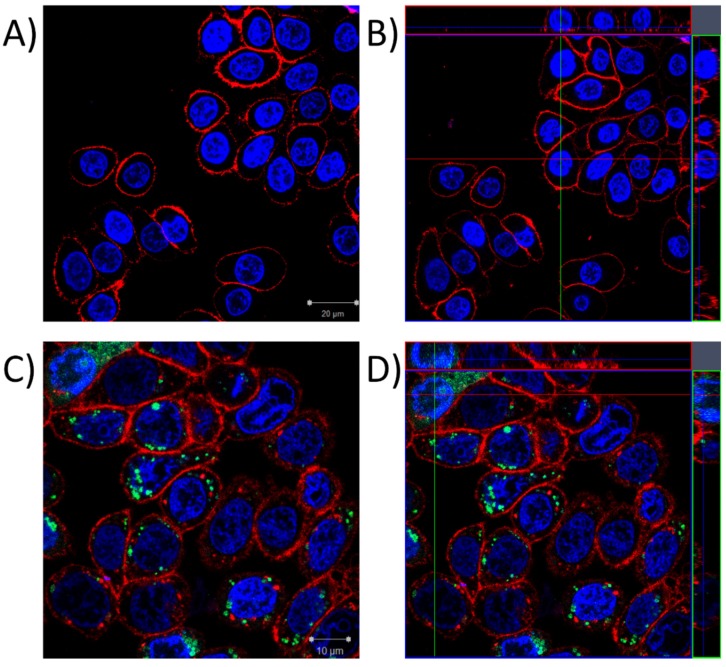
Confocal microscopy images of HT-29 cells, incubated with a solvent control (**A**,**B**) or 10 µM P8-D6 (**C**,**D**), with red staining for plasma membranes, blue for nuclear structures, and green as the auto-fluorescence of P8-D6. (**A**) and (**C**) show the central layer of the z-stacked structured illumination microscope (SIM) images, (**B**) and (**D**) shows a rendering of the images including the orthogonal cut view, where the nuclear residence of P8-D6 is visible in the top left corner of 4D.

To further investigate the question of whether P8-D6 enters the nucleus or accumulates in the perinuclear region, confocal microscopy experiments were conducted. The results supported the above reported results of the microplate reader experiments, showing cellular uptake of the compound (green fluorescence) and accumulation mainly close to the nuclei (Figure 4C). The 3D image analysis revealed the localization of a small proportion of the applied P8-D6 inside the nucleus, visible as intranuclear green dots in the orthogonal cuts (Figure 4D).

### 2.2. Stabilization of Topo–DNA Complexes

**Figure 5:**
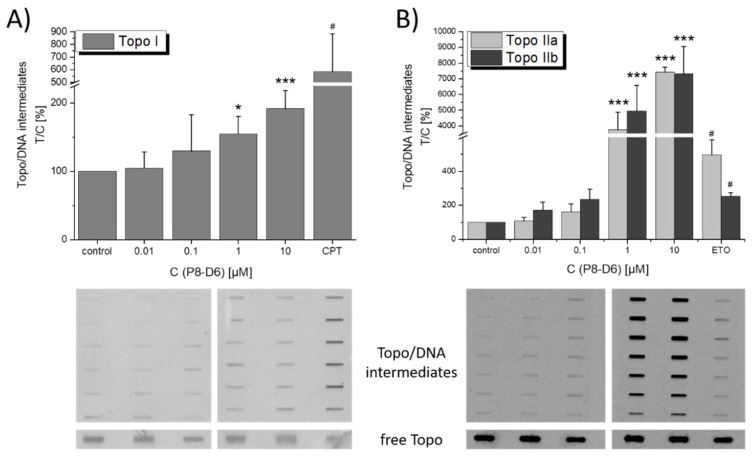
Stabilization of cleavable complexes in HT-29 cells after 1 h incubation with P8-D6, as measured with the ICE assay for (**A**) Topo I, compared to camptothecin (50 µM), and (**B**) Topo IIa and IIb, compared to etoposide (50 µM). Graphs show the results of at least five independent experiments, expressed as mean + SD and relative to the solvent control. Below the graphs, images of representative membranes are displayed. Significant differences were calculated by one-way ANOVA, followed by Fisher’s LSD test. “*”, “**”, and “***” indicate a significant difference to the respective no-effect level with *p* < 0.05, 0.01, and 0.001, respectively. The significant difference between solvent and positive control was calculated using Student’s *t*-test (*p* < 0.05) and is indicated with “#”.

After treating HT-29 cells for 1 h, P8-D6 was found to stabilize cleavable complexes of DNA with Topo I and with both Topo II isoforms in the “in vivo complex of enzyme” (ICE) assay. Regarding Topo I, P8-D6 increased the amount of detectable Topo–DNA intermediates at concentrations ≥1 µM (Figure 5A). The highest applied concentration (10 µM) enhanced the level of covalent DNA–Topo intermediates by 200% as compared to the solvent control (1% (*v*/*v*) DMSO), but stayed well below the increase caused by our positive control (50 µM camptothecin; ~600%). A non-significant stabilization of Topo II–DNA complexes was visible at concentrations as low as 0.01 µM P8-D6. However, due to the massive induction and corresponding standard deviations, this trend was significant for concentrations ≥1 µM only (Figure 5A). The induction exceeded by far the Topo-poisoning properties of our positive control, the chemotherapeutic agent etoposide (50 µM). P8-D6 apparently showed no preference for a particular isoform of the enzyme in HT-29 cells.

### 2.3. Genotoxicity

**Figure 6:**
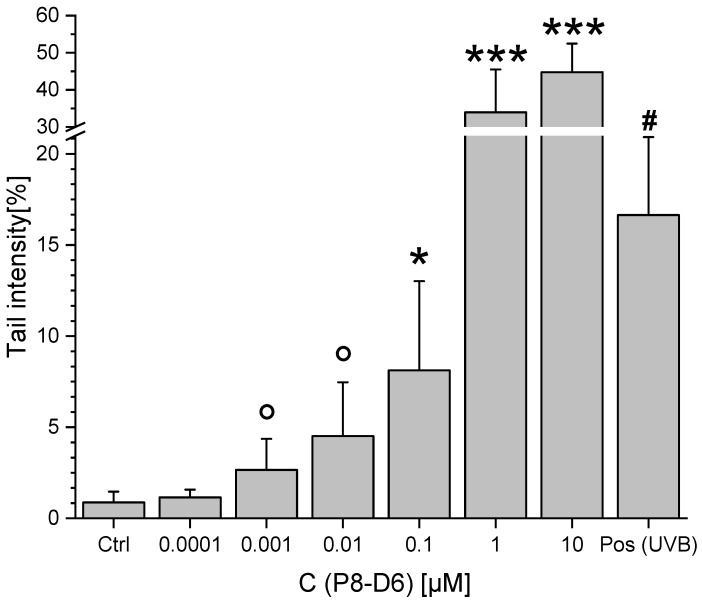
The induction of DNA strand breaks, measured in HT-29 cells by comet assay after 1 h incubation with P8-D6. Graphs show the tail intensity (a measure of genotoxicity) as mean + SD of at least five independent experiments, with UV-B radiation as positive control. Significant differences to the solvent control were calculated by one-way ANOVA followed by Fisher’s LSD test, and are indicated with “*” (*p* < 0.05), “**” (*p* < 0.01), and “***” (*p* < 0.001), respectively. The values marked with “°” were not significantly different from the control when ANOVA was conducted with the full data, but became significant when the two highest concentrations of P8-D6 were excluded due to the high deviations at those concentrations. The significant difference between solvent and positive control was calculated using Student’s *t*-test (*p* < 0.05) and is indicated with “#”.

An incubation of HT-29 cells with P8-D6 for 1 h caused a concentration-dependent increase of “tail intensities” in the comet assay, as a measure for the induction of DNA strand breaks. This genotoxic trend started at concentrations as low as 1 nM and reached significant levels for concentrations ≥100 nM (Figure 6). When excluding the two highest concentrations from the statistical calculation due to their extreme genotoxicity and thus high deviations, the induced DNA damage was significantly different to the damage of the solvent control at 1 nM and 10 nM as well. 

## 3. Discussion

Previously, we documented the dual inhibitory potential of P8-D6 against Topo I and II under cell-free conditions, as well as its strong cytotoxic effects on multiple cell lines [5]. However, the mechanism of action was not fully elucidated, leaving open research questions to be addressed.

Thus, in the present study, we assessed the mechanistic behavior of the compound in HT-29 colon carcinoma cells. Fluorescence and confocal microscopy allowed the cellular uptake of P8-D6, enrichment in the perinuclear region (Figure 2, Figure 3 and Figure 4), and partial localization within the nucleus (Figure 4D) to be modeled. These results allowed the conclusion to be drawn that within cells, P8-D6 may indeed reach its potential nuclear targets, Topo I and II. Nevertheless, for therapeutic applications, the mode of interaction with the target enzyme is of utmost importance to achieve specificity and effectiveness. The ICE assay allows discernment between unbound Topo and the proportion of Topo which is covalently linked to the DNA [16]. Topo poisons are expected to enhance the amount of Topo trapped in cleavable complexes with DNA [17]. For example, the approved chemotherapeutic drugs topotecan and camptothecin effectively inhibit Topo I by forming a Topo–DNA intermediate, while etoposide forms this cleavable complex with Topo II [16,18,19,20]. The importance of topoisomerase poisons in cancer therapy is demonstrated by their various applications, such as treatment of ovarian cancer, colon carcinoma, lung cancer, soft tissue sarcoma, or leukemia by topotecan, irinotecan, and etoposide [11,21].

Our results demonstrated that P8-D6 enhances the level of these DNA–Topo intermediates for both Topo I and the Topo II isoforms at concentrations ≥1 µM (Figure 5), characterizing the compound as a dual Topo poison not only under cell-free conditions but also in human tumor cells. Considering the impact achieved on the intracellular levels of the covalent DNA–Topo intermediates, direct comparison with the Topo I poison camptothecin is difficult because of the differing concentrations, but one might speculate that P8-D6 is of similar or slightly weaker potency. Interestingly, P8-D6 showed higher potency for Topo poisoning in HT-29 cells compared to the known Topo II poison etoposide (ETO), with a 15-fold increase in cleavable complexes containing Topo IIα and a 29-fold increase for Topo IIβ, at just a fifth of the concentration (10 µM P8-D6 vs. 50 µM ETO). As ETO is a chemotherapeutic drug already approved and widely used for treatment of small-cell lung cancer and testicular cancer [22], the superior efficiency of our test compound towards Topo II inhibition seems very promising for further development. 

Another confirmation for the toxic potency of P8-D6 was established by measuring its genotoxic properties. Effective poisoning of Topo is expected to result in substantial DNA strand breaks, a key event for the antineoplastic properties of respective compounds. Established substances such as topotecan or etoposide have already demonstrated these important effects for tumor cell activity [23]. In accordance with the results of the ICE assay, P8-D6 showed potent DNA-damaging properties in the comet assay (1 h incubation, ≥1 nM, Figure 6).

In general, selective inhibition of topoisomerase I results in an increased activity of topoisomerase II and vice versa [24]. Thus, a major advantage of a dual topoisomerase inhibitor is the avoidance of certain resistance developments. Although no dual Topo inhibitor is currently approved for therapeutic use, the effect of cleavable complex stabilization and genotoxicity of this mechanism of action has been investigated for substances such as pyrazoloacridine, intoplicine, or TAS-103 [25,26,27,28]. These substances showed a promising outcome on human cancer but clinical trials were stopped due to neutropenia, hepatotoxicity, or anemia [29,30]. However, our compound belongs to the benzo[*c*]phenanthridine class, and thus exhibits different biological activities than similar potential chemotherapeutics like indolyl quinoline or acridine derivatives. This might partially be attributed to P8-D6 being more hydrophilic as compared to dual topoisomerase inhibitors already tested in clinical phase I studies. Thus, the compound should be considered a promising alternative to existing dual Topo inhibitors for clinical application. 

The in vivo toxicity study on athymic nude mice indicated reversible toxic effects at a concentration of 5 mg P8-D6 kg^−1^ body weight [5]. Adverse hepatotoxic effects are currently being investigated in vitro. The study at hand provides the mechanistic elucidation for P8-D6′s activity; thus, the last remaining step in the extensive in vivo testing that is planned to be conducted in further surveys examining the effect of the chemical in various entities. In addition to tumor reduction, there is a major focus in these studies on examining organ toxicity. 

## 4. Materials and Methods 

### 4.1. Chemicals, Antibodies, and Enzymes

P8-D6 was synthesized as recently described [31]. The following antibodies, obtained from Santa Cruz (Heidelberg, Germany), were used for our experiments: anti-Topo I (sc-10783, rabbit polyclonal IgG, lot #D414), anti-Topo IIα (sc-5346, goat polyclonal IgG, lot #G2611), anti-Topo IIβ (sc-13059, rabbit polyclonal IgG, lot #D2214), anti-rabbit (sc-2357, mouse polyclonal IgG, HRP-coupled, lot #B0817), and anti-goat (sc-2354, mouse polyclonal IgG, HRP-conjugated, lot #H2213). ECL detection reagent and nitrocellulose membranes were ordered from GE Healthcare (Buckinghamshire, UK). Agarose was purchased from Bio-Rad (Vienna, Austria), and ethidium bromide and etoposide from Sigma-Aldrich (Taufkirchen, Germany). Live Cell Imaging Solution (LCIS) and all media and supplements for cell culture were obtained from GIBCO Invitrogen (Karlsruhe, Germany).

### 4.2. Cell Culture and Treatment

The HT-29 human colon carcinoma cell line was obtained from DSMZ (Braunschweig, Germany) and grown in Dulbecco’s modified Eagle’s medium (DMEM), supplemented with 10% (*v*/*v*) heat-inactivated fetal calf serum and 1% (*v*/*v*) penicillin/streptomycin. Media and supplements were purchased from Invitrogen^TM^ Life Technologies (Karlsruhe, Germany). Cell cultivation was performed at 37 °C, with 5% CO_2_ and under humified conditions. Cells were routinely tested for the absence of mycoplasm contamination. For incubations, P8-D6 was dissolved in DMSO and added to the incubation medium, resulting in a solvent concentration of 1% (*v*/*v*) DMSO plus final concentrations of the compound between 0.1 nM and 10 µM.

### 4.3. Fluorescence Imaging

In preliminary experiments, P8-D6 was found to possess fluorescent properties at an emission maximum of 530 nm. To confirm the ability of P8-D6 to penetrate cell nuclei, 8000 cells/well were seeded in black 96 well plates and allowed to grow for 48 h. Cells were then washed with pre-warmed PBS and incubated for 1 h with different concentrations of P8-D6 and a respective solvent control (1% *v*/*v* DMSO) diluted in live cell imaging solution (LCIS). Subsequently, cells were rinsed with PBS and nuclei counterstained with a “Hoechst 33258” solution containing 10 µL/mL pentahydrate (bis-benzimide) for 20 min. Next, staining solutions were discarded and cells were washed with LCIS. 100 µL/well LCIS was added and fluorescence imaging was performed using DAPI (377.4 nm/447 nm) and GFP (469.5 nm/525 nm) filters with a BioTek^TM^ Cytation^TM^3 Cell Imaging Multi-Mode Microplate Reader and the Gen5 software.

### 4.4. Confocal Microscopy

A total 1 × 10^5^ cells were seeded in CELLview^TM^ culture dishes and allowed to grow for 48 h, following a 1 h incubation with the test compounds or the solvent control (1% *v*/*v* DMSO) diluted in LCIS (supplemented with 10% FCS). Cells were washed with DPBS and then stained for 20 min with a mixed staining solution containing Hoechst 33258 and CellMask^TM^ deep red. Subsequently, cells were washed twice with PBS, and clean LCIS was added to the dishes for microscopy. Confocal microscopy was conducted with a Confocal LSM Zeiss 710 equipped with the ELYRA PS.1 system, a Plan-Apochromat 63×/1.4 oil objective (zoom 2×) using three lasers. P8-D6 was detected at 488 nm (green), cell membranes at 647 nm (CellMask^TM^, deep red), and nuclei at 405 nm (DAPI, blue).

For image analysis, random cells were selected using only the DAPI channel to exclude a biased cell selection. Afterwards, 3D images were reconstructed using the Zeiss Zen software.

### 4.5. In Vivo Complex of Enzyme (ICE) Assay

ICE assay experiments were carried out as previously described by Subramanian et al. [16]. In brief, Petri dishes (diameter 10 cm) were used to grow 6.5 × 10^6^ HT-29 cells for 48 h, followed by 1 h incubation with different concentrations of P8-D6 or respective controls. Subsequently, cells were washed with PBS, lysed, and harvested. The lysate was put on a density gradient (0.75–1.6 mg/mL cesium chloride) and centrifuged at 100,000 *g* for 20 h to separate the heavier Topo–DNA complexes from the lighter free Topos. Fractions of the gradient were blotted on a nitrocellulose membrane, which were after blocking treated with HRP-coupled antibodies to later detect Topo I and both Topo II isoforms using chemoluminescence. Pictures of the membranes were captured with a LAS-4000 imager (Fujifilm Life Science, Cleve, Germany).

### 4.6. Single Cell Gel Electrophoreses (“Comet Assay”)

The comet assay was performed according to the guidelines of Tice et al. [32], as recently described [33]. Briefly, 1.5 × 10^5^ HT-29 cells were seeded in Petri dishes (diameter 3.5 cm). After 48 h growth in complete medium, cells were incubated with different concentrations of P8-D6 and the solvent control (1% (*v*/*v*) DMSO) in FCS-free DMEM for 1 h. To gain a positive control, one Petri dish incubated with 1% (*v*/*v*) DMSO was subjected to UV-B radiation (31.2 J, 1 min). Afterwards, all dishes were washed twice with PBS and the cells were harvested using trypsin. Cell viability was checked using trypan blue exclusion. For each sample, an object slide with two agarose pads was prepared, each containing 30,000 embedded cells. After lysis by using a buffer containing Triton X and lauroyl sarcosinate, the slides were washed with PBS and subsequently equilibrated in alkalic electrophoresis buffer (pH 13) for 20 min at 0 °C. Electrophoresis was carried out for 20 min at 25 V, 300 ± 3 mA, 0 °C. Afterwards, slides were neutralized and stained with ethidium bromide (0.02 g/mL in water). Subsequently, microscopy was carried out using a Zeiss Axioskop (λ_ex_ = 546 nm; λ_em_ = 590 nm) and obtained images were analyzed using the “Comet Assay IV” software (Perceptive Instruments, Suffolk, UK) to score the tail intensity of 100 nuclei per slide.

## 5. Conclusions

Taken together, the results of the present study demonstrated that P8-D6 acts as a dual Topo poison in human colon cancer cells. The observed potency for the stabilization of the DNA–Topo intermediate and for DNA breakage supports the hypothesis that dual Topo poisoning plays a central role in the previously observed potent antineoplastic activity of P8-D6 in vitro, and demonstrates the compound’s high potential for chemotherapeutic use. 

## References

[1] Dvorak Z., Kubáň V., Klejdus B., Hlaváč J., Vičar J., Ulrichová J., Simánek V. (2006). Quaternary benzo[c]phenanthridines sanguinarine and chelerythrine: A review of investigations from chemical and biological studies. Heterocycles.

[2] El-Readi M.Z., Eid S., Ashour M.L., Tahrani A., Wink M. (2013). Modulation of multidrug resistance in cancer cells by chelidonine and Chelidonium majus alkaloids. Phytomedicine.

[3] Larsen A.K., Grondard L., Couprie J., Desoize B., Comoe L., Jardillier J.C., Riou J.F. (1993). The antileukemic alkaloid fagaronine is an inhibitor of DNA topoisomerases I and II. Biochem. Pharmacol..

[4] Meier C., Kotthaus J., Stenzel L., Girreser U., Heber D., Clement B. (2012). Synthesis and physicochemical characterization of novel 6-aminopyrido[3,4-*c*][1,9]phenanthrolines as aza-analogs of benzo[c]phenanthridines. Tetrahedron.

[5] Meier C., Steinhauer T.N., Koczian F., Plitzko B., Jarolim K., Girreser U., Braig S., Marko D., Vollmar A.M., Clement B. (2017). A Dual Topoisomerase Inhibitor of Intense Pro-Apoptotic and Antileukemic Nature for Cancer Treatment. ChemMedChem.

[6] Pommier Y., Sun Y., Huang S.N., Nitiss J.L. (2016). Roles of eukaryotic topoisomerases in transcription, replication and genomic stability. Nat. Rev. Mol. Cell Biol..

[7] Nitiss J.L. (2009). DNA topoisomerase II and its growing repertoire of biological functions. Nat. Rev. Cancer.

[8] McClendon A.K., Osheroff N. (2007). DNA topoisomerase II, genotoxicity, and cancer. Mutat. Res..

[9] Larsen A.K., Escargueil A.E., Skladanowski A. (2003). Catalytic topoisomerase II inhibitors in cancer therapy. Pharmacol. Ther..

[10] Nitiss J.L. (2009). Targeting DNA topoisomerase II in cancer chemotherapy. Nat. Rev. Cancer.

[11] Pommier Y. (2006). Topoisomerase I inhibitors: Camptothecins and beyond. Nat. Rev. Cancer.

[12] Salerno S., Da Settimo F., Taliani S., Simorini F., La Motta C., Fornaciari G., Marini A.M. (2010). Recent advances in the development of dual topoisomerase I and II inhibitors as anticancer drugs. Curr. Med. Chem..

[13] Esselen M., Boettler U., Teller N., Bachler S., Hutter M., Rufer C.E., Skrbek S., Marko D. (2011). Anthocyanin-rich blackberry extract suppresses the DNA-damaging properties of topoisomerase I and II poisons in colon carcinoma cells. J. Agric. Food Chem..

[14] Esselen M., Barth S.W., Winkler S., Baechler S., Briviba K., Watzl B., Skrbek S., Marko D. (2013). Anthocyanins suppress the cleavable complex formation by irinotecan and diminish its DNA-strand-breaking activity in the colon of Wistar rats. Carcinogenesis.

[15] Aichinger G., Puntscher H., Beisl J., Kutt M.L., Warth B., Marko D. (2018). Delphinidin protects colon carcinoma cells against the genotoxic effects of the mycotoxin altertoxin II. Toxicol. Lett..

[16] Subramanian D., Furbee C.S., Muller M.T. (2001). ICE bioassay. Isolating in vivo complexes of enzyme to DNA. Methods Mol. Biol..

[17] Nitiss J.L., Soans E., Rogojina A., Seth A., Mishina M. (2012). Topoisomerase Assays. Curr. Protoc. Pharmacol..

[18] Patel A.G., Flatten K.S., Peterson K.L., Beito T.G., Schneider P.A., Perkins A.L., Harki D.A., Kaufmann S.H. (2016). Immunodetection of human topoisomerase I-DNA covalent complexes. Nucleic Acids Res..

[19] Gromova I.I., Kjeldsen E., Svejstrup J.Q., Alsner J., Christiansen K., Westergaard O. (1993). Characterization of an altered DNA catalysis of a camptothecin-resistant eukaryotic topoisomerase I. Nucleic Acids Res..

[20] Subramanian D., Kraut E., Staubus A., Young D.C., Muller M.T. (1995). Analysis of topoisomerase I/DNA complexes in patients administered topotecan. Cancer Res..

[21] Baldwin E.L., Osheroff N. (2005). Etoposide, topoisomerase II and cancer. Curr. Med. Chem. Anticancer Agents.

[22] Pommier Y., Leo E., Zhang H., Marchand C. (2010). DNA topoisomerases and their poisoning by anticancer and antibacterial drugs. Chem. Biol..

[23] Swift L.H., Golsteyn R.M. (2014). Genotoxic anti-cancer agents and their relationship to DNA damage, mitosis, and checkpoint adaptation in proliferating cancer cells. Int. J. Mol..

[24] van Gijn R., Lendfers R., Schellens J., Bult A., Beijnen J. (2000). Dual topoisomerase I/II inhibitors. J. Oncol. Pharm. Pract.

[25] Poddevin B., Riou J.F., Lavelle F., Pommier Y. (1993). Dual topoisomerase I and II inhibition by intoplicine (RP-60475), a new antitumor agent in early clinical trials. Mol. Pharmacol..

[26] Kluza J., Lansiaux A., Wattez N., Mahieu C., Osheroff N., Bailly C. (2000). Apoptotic response of HL-60 human leukemia cells to the antitumor drug TAS-103. Cancer Res..

[27] Adjei A.A., Charron M., Rowinsky E.K., Svingen P.A., Miller J., Reid J.M., Sebolt-Leopold J., Ames M.M., Kaufmann S.H. (1998). Effect of pyrazoloacridine (NSC 366140) on DNA topoisomerases I and II. Clin. Cancer Res..

[28] Padget K., Stewart A., Charlton P., Tilby M.J., Austin C.A. (2000). An investigation into the formation of N- [2-(dimethylamino)ethyl]acridine-4-carboxamide (DACA) and 6-[2-(dimethylamino)ethylamino]- 3-hydroxy-7H-indeno[2,1-C]quinolin-7-one dihydrochloride (TAS-103) stabilised DNA topoisomerase I and II cleavable complexes in human leukaemia cells. Biochem. Pharmacol..

[29] Newman R.A., Kim J., Newman B.M., Bruno R., Bayssas M., Klink-Alaki M., Pazdur R. (1999). Phase I trial of intoplicine (RP 60475) administered as a 72 h infusion every 3 weeks in patients with solid tumors. Anti-Cancer Drugs.

[30] Ramaswamy B., Mrozek E., Kuebler J.P., Bekaii-Saab T., Kraut E.H. (2011). Phase II trial of pyrazoloacridine (NSC#366140) in patients with metastatic breast cancer. Investig. New Drugs.

[31] Clement B., Girreser U., Steinhauer T.N., Meier C., Marko D., Aichinger G., Kaltefleiter I., Stenzel L., Heber D., Weide M. (2016). 11-Substituted Benzo c phenanthridines: New Structures and Insight into Their Mode of Antiproliferative Action. ChemMedChem.

[32] Tice R.R., Agurell E., Anderson D., Burlinson B., Hartmann A., Kobayashi H., Miyamae Y., Rojas E., Ryu J.C., Sasaki Y.F. (2000). Single cell gel/comet assay: Guidelines for in vitro and in vivo genetic toxicology testing. Environ. Mol. Mutagen..

[33] Aichinger G., Beisl J., Marko D. (2017). Genistein and delphinidin antagonize the genotoxic effects of the mycotoxin alternariol in human colon carcinoma cells. Mol. Nutr. Food Res..

